# New hosts for a snake's helminth: First report of intermediate and definitive hosts naturally infected by *Ophidascaris arndti* (Ascarididae) in the wild

**DOI:** 10.1016/j.ijppaw.2022.11.003

**Published:** 2022-11-14

**Authors:** Raquel de Oliveira Simões, Beatriz Elise de Andrade-Silva, Thiago dos Santos Cardoso, Rosana Gentile, Jonathan Gonçalves-Oliveira, Roberto do Val Vilela, Arnaldo Maldonado Júnior

**Affiliations:** a Departamento de Parasitologia Animal, Universidade Federal Rural do Rio de Janeiro, Seropédica, RJ, Brazil; b Laboratório de Biologia e Parasitologia de Mamíferos Silvestre Reservatório, Instituto Oswaldo Cruz, Fundação Oswaldo Cruz, Rio de Janeiro, RJ, Brazil; c Programa de Pós-graduação em Biologia Parasitária, Instituto Oswaldo Cruz, Fundação Oswaldo Cruz, Rio de Janeiro, RJ, Brazil; d Programa de Pós-Doutorado Nota 10 – 2021, FAPERJ – Fundação Carlos Chagas Filho de Amparo à Pesquisa do Estado do Rio de Janeiro, Av. Erasmo Braga, 118, 20020-000, Rio de Janeiro, RJ, Brazil; e Laboratory of Zoonotic Vector-borne diseases, The Koret School of Veterinary Medicine, The Hebrew University of Jerusalem, Israel

**Keywords:** Viperidae, Cricetidae, Morphology, Phylogenetics, Mitochondrial cytochrome *c* oxidase, Atlantic forest

## Abstract

We wish to report the occurrence of adult nematodes *Ophidascaris arndti* (Ascarididae) naturally infecting a new definitive host, the Fonseca's lancehead *Bothrops fonsecai* (Viperidae), and third-stage larvae of *O. arndti* parasitizing a new intermediate host, the montane grass mouse *Akodon montensis* (Cricetidae), both found in the Atlantic Forest of the state of Rio de Janeiro, Brazil. We elucidated the morphological characteristics of both adults and larvae using light and scanning electron microscopy (SEM). Taxonomic affinities between larvae and adult worms were assessed using MT-CO1 gene sequences. Adult and larval gene sequences formed a well-supported clade and had low pairwise p-distances, suggesting that they are conspecific. Our phylogenies also supported the ‘arndti’, ‘filaria’, and ‘obconica’ groups as independent lineages and confirmed the allocation of *Ophidascaris* within the family Ascarididae, although as an early offshoot. This is the first report of natural infection of this helminth's larvae in a wild intermediate host.

## Highlights

• *Ophidascaris arndti* found infecting new rodent and snake species in the wild.
• L_3_ larvae found in a grass mouse pointing to its role as an intermediate host.
• Adult worms found in a Fonseca's Lancehead, adding a new definitive host species.
• Molecular analyses confirmed the closeness between mouse and snake helminths.
• Phylogenies supported ‘arndti’, ‘filaria’, and ‘obconica’ groups as independent.

## Graphical abstract

**Figure:**
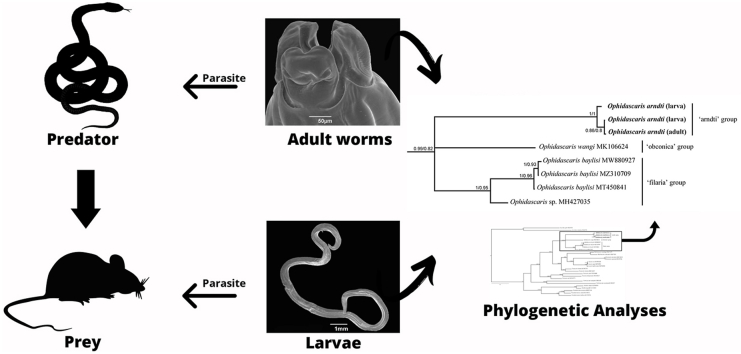

## 1 Introduction

The genus *Ophidascaris* Baylis, 1921 is placed in the family Ascarididae Baird, 1853, subfamily Angusticaecinae Skrjabin and Karokhun, 1945 (Anderson et al., 2009). This genus currently comprises more than 22 recognized species parasitizing reptiles, mostly snakes (Sprent, 1988; Li et al., 2014). Sprent (1988) revised the genus, dividing it into five specific groups: ‘arndti’, ‘filaria’, ‘najae’, ‘obconica’, and ‘radiosa’. This classification considered the vertebrate host, geographical distribution, and morphological features. According to the Key to species groups in *Ophidascaris* provided by Sprent (1988), the ‘arndti’ species group is characterized by having lateral alae inconspicuous; in male postcloacal rough area absent and spicules less than twice length of ejaculatory duct. The ‘arndti’ group encompasses the following species: *O. arndti* Sprehn (1929) (syn: *Ascaris quadrangular* Schneider, 1866; *O. travassosi* Vaz, 1938; *O. sprenti* Araujo, 1969); *O. ochoteranai* Caballero, 1939 (*species inquirenda*); and *O. sicki* (syn: *O. arndti* referred by Freitas, 1955; *O. cretinorum* Freitas, 1968). These species occur in South American viperids and colubrids.

Adult forms are found in the stomach or small intestine in definitive hosts, mainly snakes (Freitas, 1968; Pinto et al., 2010). Their life cycle is heteroxenous, with amphibians and rodents as intermediate hosts infected by ingesting eggs with L_2_ larvae (Freitas, 1968; Araujo and Machado, 1980; Anderson, 2000). Snakes become infected after eating intermediate hosts parasitized by L_3_ larvae encapsulated in the muscles or viscera (Araujo and Machado, 1980; Anderson, 2000). The adults fix deeply in the submucosa promoting a focal ulceration lesion (Jacobson, 2007), characterized as necro-ulcerative gastroenteritis (Anderson, 2000; Mello et al., 2017). Moreover, they can cause mechanical obstruction or perforations of the viscera (Wilson and Carpenter, 1996; Jacobson, 2007). All preceding information on the life cycles and hosts of some *Ophidascaris* species (such as *O. trichuriformis*) is based on experimental infections only. No life cycle of *Ophidascaris* has been previously elucidated from natural infection.

In this study, we reported, for the first time, adult worms of *O. arndti* naturally infecting the Fonseca's lancehead, *Bothrops fonsecai* Hoge and Belluomini, 1959 (Viperidae), and third-stage larvae parasitizing the montane grass mouse, *Akodon montensis* Thomas, 1913 (Cricetidae), both collected in the Atlantic Forest of the state of Rio de Janeiro, Brazil. We elucidated some morphological characteristics of adults and larvae using light and scanning electron microscopy (SEM). To confirm that adult worms and larvae belonged to the same species and their taxonomic affinities, we conducted molecular phylogenetic analyses using novel sequences of the mitochondrial cytochrome-c oxidase subunit I (MT-CO1) gene*.*

## 2 Material and methods

### 2.1 Collection and identification of hosts and helminths

Third-stage larvae of *O. arndti* were retrieved from the musculature of two individuals of the sigmodontine rodent *A. montensis.* The rodents were trapped during a comprehensive study of biodiversity that aimed to survey the Atlantic Forest fauna in preserved areas. One of the rodents was captured in a Tomahawk trap placed on the ground (22°30′12.1″ S 43°07′08″ W) and the other in a pitfall trap (22°30′21.7″ S 43°06′50″ W), both in areas of montane dense ombrophilous Atlantic Forest in the Serra dos Órgãos National Park (PARNASO), municipality of Petrópolis, state of Rio de Janeiro. The rodents were euthanized, necropsied for helminth recovery and other studies, taxidermized, identified by external and cranial morphology and by karyotyping, and deposited at the Museu Nacional, Universidade Federal do Rio de Janeiro (MN/UFRJ). The animals were captured under the authorization issued by the Brazilian Ministry of the Environment's Instituto Chico Mendes de Conservação da Biodiversidade (SISBio, license number 45839–1). All procedures followed the guidelines for capture, handling, and care of animals of the Ethical Committee on Animal Use of the Oswaldo Cruz Foundation (CEUA license number LW – 39/14). Biosafety techniques and personal safety equipment were used during all procedures involving animal handling and biological sampling. Adult nematodes were recovered from the stomach of a road-killed individual of *B. fonsecai*, at the BR 040 highway in the municipality of Teresópolis, state of Rio de Janeiro.

### 2.2 Morphological analyses

The rodents and the snake were examined for the presence of helminths. Adult worms were clarified in 50% alcohol-glycerol and mounted on temporary slides. Mid-body sections of the adult worms and two L3 larvae were preserved in 70% ethanol for molecular analyses. Morphological analyses were conducted using an Olympus BX-51 light microscope. Images were captured using an Olympus DP-12 digital camera. Drawings were made using a drawing tube attached to a Nikon Y-IDT light microscope. A range of measurements was taken in millimeters. Measurements were based on nine specimens, four males and five females. However, due to the poor state of preservation of the material, some morphological structures were not observed in all specimens. Nematode identification was performed according to Freitas (1968), Vicente et al. (1993), Anderson et al. (2009), and specific articles (Sprehn, 1929; Vaz, 1938; Araujo, 1969; Sprent, 1988). Vouchers were deposited in the Helminthological Collection of the Oswaldo Cruz Institute (CHIOC) (CHIOC Nº XXXX), Oswaldo Cruz Foundation (Fiocruz).

For scanning electron microscopy (SEM) analysis, nematodes were washed in 0.1 M Na-cacodylate buffer, pH 7.2, post-fixed in 1% OsO_4_ and 0.8% K_3_Fe (CN)_6_, dehydrated in graded ethanol (30–100%) for 2 h, and dried by the critical point method with CO_2_ (CPD 030, Balzers, Switzerland). The samples were mounted on aluminum stubs, coated with a 20 nm layer of gold, and examined with a Jeol JSM 6390LV scanning electron microscope (operating at 15 kV) at the Rudolf Barth Electron Microscopy Platform of the Oswaldo Cruz Institute, FIOCRUZ.

### 2.3 DNA isolation, amplification, and sequencing

Total genomic DNA was isolated from nematode specimens of *O. arndti* (one adult and two larvae) using the Qiagen QIAamp DNA Mini Kit, according to the manufacturer's protocol. DNA amplification by polymerase chain reaction was conducted using the primer cocktail, described by Prosser et al. (2013), for the barcode region of the mitochondrial cytochrome-c oxidase subunit I (MT-CO1) gene. Reactions were carried out in a total volume of 25 μl containing 12.5 μl of PCR Master Mix (Promega Corporation), 0.5 μl of each primer cocktail, 1 μl of DNA, and ultrapure water. The cycling conditions were 94 °C for 1 min; five cycles at 94 °C for 40 s, 45 °C for 40 s, and 72 °C for 1 min; 35 cycles at 94 °C for 40 s, 51 °C for 40 s, and 72 °C for 1 min; and a final extension at 72 °C for 5 min. Successfully amplified products were purified using the QIAquick PCR Purification Kit (Qiagen), following the manufacturer's protocol.

Sequencing reactions were performed using the Big Dye Terminator v3.1 Cycle Sequencing Kit (Applied Biosystems, USA) on both strands, using each primer of the cocktail mentioned previously separately. Reactions, cycle-sequenced product precipitation, formamide resuspension, and DNA sequencing were conducted at the capillary electrophoresis DNA sequencing (96 capillaries) - 3730xL platform of the Oswaldo Cruz Institute, FIOCRUZ (https://plataformas.fiocruz.br/). Electropherograms were assembled into contiguous and edited for errors and ambiguities using the Geneious Prime 2022.2.1 software platform (https://www.geneious.com), resulting in consensus sequences.

Nucleotide sequence data reported in this paper are available in the GenBank database under the accession numbers: **OP256424** (larva), **OP256425** (larva), and **OP256426** (adult).

### 2.4 Phylogenetic analyses

We employed mitochondrial cytochrome *c* oxidase I (MT-CO1) gene sequences for phylogenetic analyses. The dataset consisted of 29 taxa, including sequences we generated and those available in GenBank, representatives of different ascaridoid families. As an outgroup, we added sequences of heterakoid species, *Ascaridia gali* and *A. columbae*. GenBank accession numbers were appended to the taxa names in the phylogenies.

We aligned the MT-CO1 gene sequences using the TranslatorX server (Abascal et al., 2010). Initial alignment for TranslatorX was provided using the MUSCLE algorithm (Edgar, 2004). The resulting alignment was edited and trimmed of regions with poor overlap using the software Mesquite Version 3.70 (Maddison and Maddison, 2021).

Substitution saturation was assessed, as proposed by Xia et al. (2003) and Xia and Lemey (2009), using DAMBE, version 7.0.35 (Xia, 2018), in the matrix as a whole and for each codon position separately. We also tested the matrix for the presence of phylogenetic signal using the g1 statistic, examining 10,000,000 randomly generated topologies, and the PTP test, with 10,000 permutations. Both tests were implemented using PAUP* version 4.0a169 (Swofford, 2003). We also used PAUP* to calculate uncorrected pairwise genetic distances (*p* distances) between sequences.

Phylogenetic reconstruction using maximum likelihood (ML), as the optimality criterion, was carried out using the PhyML 3.0 web server (Guindon et al., 2010). The best-fit nucleotide evolutionary model was calculated under the Akaike information criterion (AIC), via SMS (Smart Model Selection) (Lefort et al., 2017). Branch supports were assessed by the approximate likelihood-ratio test (aLRT) (Anisimova and Gascuel, 2006) and by bootstrap percentages (ML-BP) after 1000 replicates.

Bayesian phylogenetic inference (BI) was carried out using MrBayes version 3.2.7a (Ronquist et al., 2012) on XSEDE using the CIPRES Science Gateway (Miller et al., 2010). Accounting for different evolutionary processes at each codon position of the MT-CO1 gene, we performed BI using distinct models per codon position, with unlinking of state frequencies and parameters. The best-fit nucleotide evolutionary model for each codon position was calculated under the Bayesian information criterion (BIC), via automated model selection (AMS) using PAUP*. Markov chain Monte Carlo (MCMC) samplings were performed for 10,000,000 generations, with four simultaneous chains, in two runs. Branch supports were assessed by Bayesian posterior probabilities (BPP), calculated from trees sampled every 1000 generations, after a 25% fraction burn-in removal. The robustness of sampling was assessed via the effective sample sizes (ESS) of parameters, calculated using Tracer v1.7.1 (Rambaut et al., 2018). After burn-in, values above 200 effectively independent samples were considered well sampled.

## 3 Results

### 3.1 Morphological description of Ophidascaris arndti

#### 3.1.1 General

**Fig. 1:**
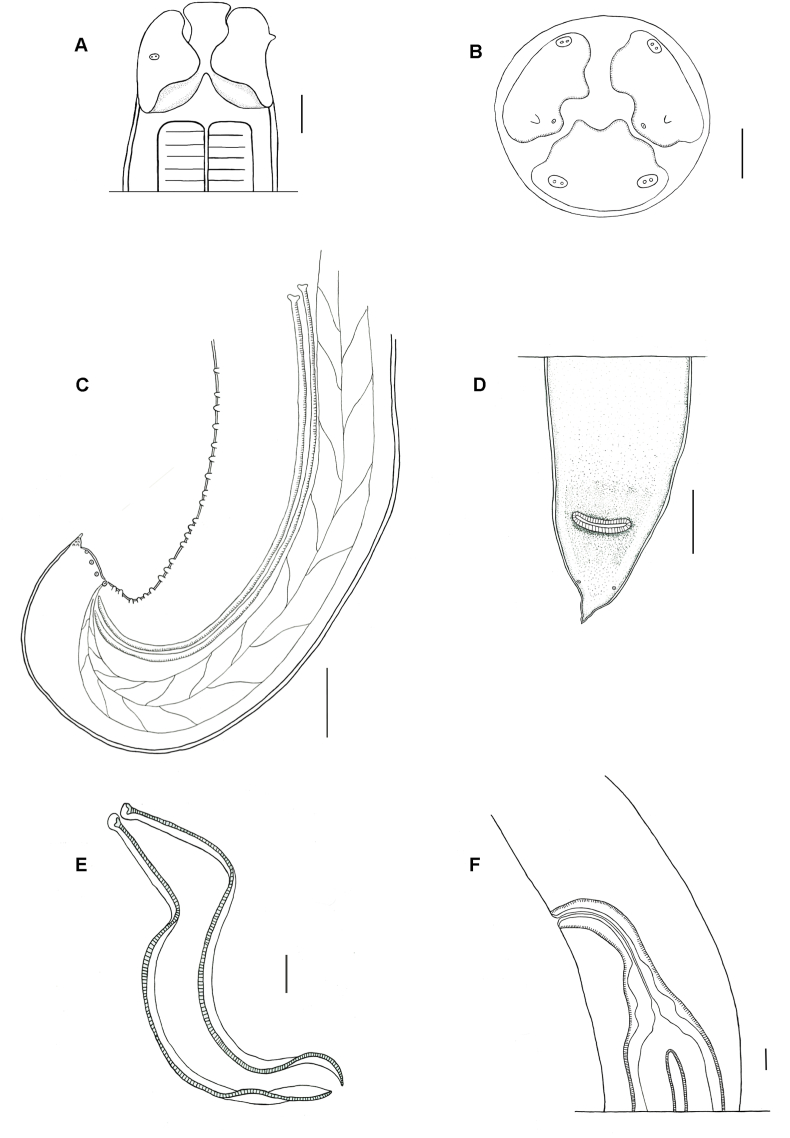
Light microscopy drawing of adult *Ophidascaris arndti* from *Bothrops freitasi*. (A) Female anterior end lateral view; (B) Female lips apical view; (C) Male posterior end lateral view, spicule, and ejaculatory duct; (D) Female posterior end ventral view; (E) Spicules; (F) Lateral view of the vulva, vagina, and uterine branches.

**Fig. 2:**
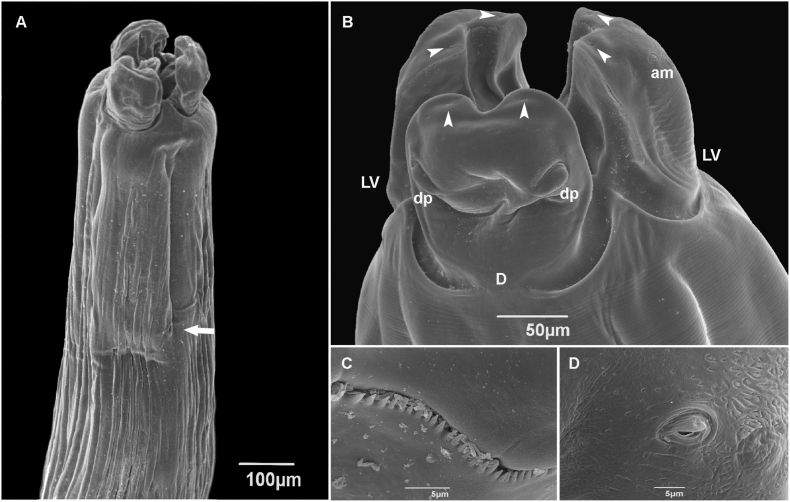
Scanning electron micrographs of *Ophidascaris arndti* from *Bothrops freitasi* adult female. (A) Anterior end lateral view showing the excretory pore (arrow); (B) Dorsal view of lips, dorsal lip (D), double papillae (dp), lateroventral lips (LV), amphids (am), pointed depression (arrowhead); (C) Detail of denticles; (D) Detailed amphids.

**Fig. 3:**
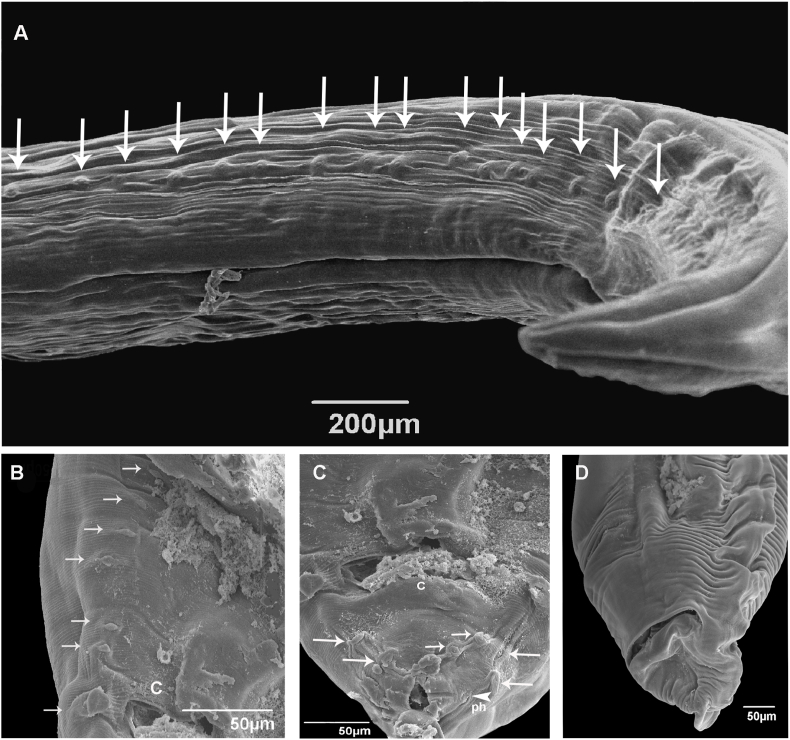
Scanning electron micrographs of *Ophidascaris arndti* from *Bothrops freitasi*: (A) Male ventral view of the posterior end papillae (arrow); (B) Male cloaca (c) and papillae (arrow); (C) Male ventral view of tail, cloaca (c), papillae (arrow), and phasmid (ph, arrowhead); (D) Female ventro-lateral view of tail.

Medium to large nematodes. Cuticle with fine longitudinal striations along the body (Fig. 2A). Anterior extremity with three slightly quadrangular lips, approximately equal in size, with deep postlabial grooves and prominent lateral membraneous flanges (Fig. 1, Fig. 2B). Presence of indentations or dentigerous ridges in the internal border, medio apical notch (Fig. 2B and C). Dorsal lip with a pair of double papillae (Fig. 1, Fig. 2B) and lateroventral lips with one double papilla, small papilla, and amphid (Fig. 1, Fig. 2D). Each lip with two pointed depressions (Fig. 2B). Presence of well-developed interlabia, triangular, approximately 1/3 length of lips (Fig. 2B). A pair of small pores present symmetrically on each lip just external to the ridge (Fig. 2B). Excretory pore situated ventrally (Fig. 2A). Male ventrolateral posterior region with row of papillae (Fig. 1, Fig. 3A). Tail of both sexes conical, female tip unornamented (Fig. 1D).

*Male:* Body length 9.13–17.77 long (N = 2), width 0.29–0.34 (N = 3). Lips 0.07–0.30 long and 0.08–0.28 wide (N = 4). Esophagus 1.07–2.06 long (N = 4). Nerve ring and excretory pore 0.48 (N = 1) and 0.31–0.47 (N = 2) from anterior end, respectively. Spicules slightly subequal, alate 1.13–1.47 (N = 3) (Fig. 1E). Difference between spicules 0.04 (N = 2). Caudal alae narrow. Presence of 22 pairs of precloacal papillae, 1 paracloacal pair, 4 postcloacal pairs, and distally small phasmids (Fig. 3A, B, and 3C). Cloaca 0.18-0-30 long (N = 3) from the posterior end. Tail 0.031–0.043 long (N = 2) (Fig. 3C).

*Female:* Body length 30.75–58.98 (N = 4), width 5.52–2.60 (N = 2). Lips 0.07–0.30 long and 0.22–0.29 wide (N = 4). Esophagus 2.17–3.46 long (N = 4). Nerve ring and excretory pore 0.38–0.42 (N = 2) and 0.56–0.58 (N = 2) from anterior end, respectively. Vulvar aperture in the anterior middle of body 10.63–11.37 long (N = 2). Uterus divided into two branches (Fig. 1F). Eggs subspherical 0.076–0.084 long, 0.06–0.07 wide (N = 5). Anus 0.40–0.46 (N = 4) from the posterior end. Tail 0.11–0.32 long (N = 4) with digitiform tip (Fig. 3D). Small lateral phasmids at the base of the tail tip (Fig. 1D).

##### 3.1.1.1 *Larvae* (L_3_): based on 4 specimens

**Fig. 4:**
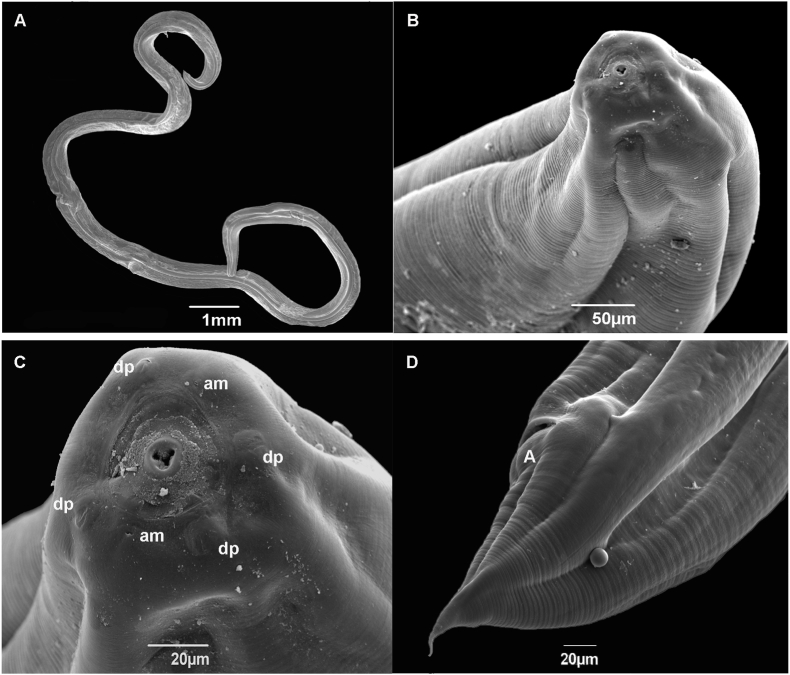
Scanning electron micrographs of *Ophidascaris arndti* larva from *Akodon montensis*. (A) Body; (B) Anterior end; (C) Apical view, amphids (am) and double papillae (dp); (D) Posterior end lateral view, anus (A).

**Fig. 5:**
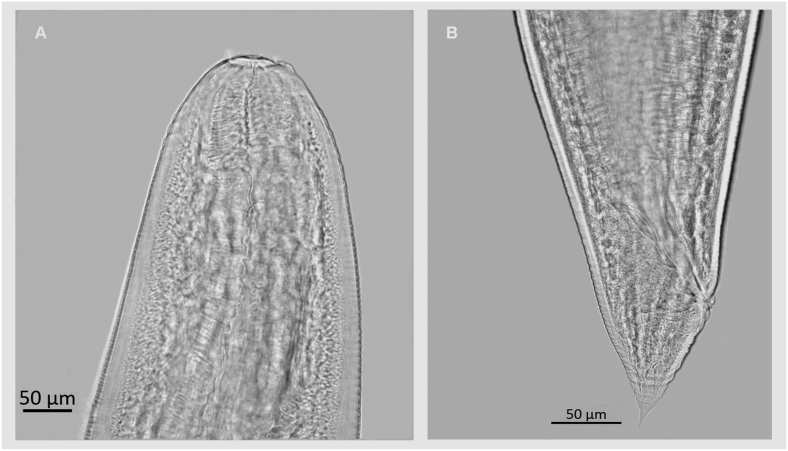
Photomicrography of *Ophidascaris arndti* larva from *Akodon montensis*. (A) Anterior end lateral view; (B) Posterior end lateral view.

Medium-size, white in color with cuticle striated transversely (Fig. 4, Fig. 5A). Body length 18.73–29.81 (N = 3) and width 0.54–0.81 (N = 3). Oral opening rounded with underdeveloped lips, four double papillae, and two amphids (Fig. 4B and C). Nerve ring and excretory pore 0.35–0.46 (N = 4), 1.16 (N = 1) from the anterior end, respectively. Esophagus length 2.17–4.26 (N = 2). Genital primordium situated at posterior third of the body 10.14 (N = 1). Anus 0.15–0.28 (N = 3) from posterior extremity. Tail 0.05–0.06 long (N = 3) (Fig. 4, Fig. 5B).

### 3.2 Molecular analyses

We successfully sequenced three nematode specimens for the MT-CO1 gene, two larvae and one adult. Alignment of the sequences in our dataset resulted, after trimming, in a matrix of 29 taxa per 702 characters, of which 444 were constant and 205 were parsimony-informative variable characters. The matrix had little substitution saturation, as conveyed by Xia's test. Saturation was also significantly negligible at the first and second codon positions, although increasing at the third codon position, from significantly low for a symmetrical tree to significantly high for an extreme asymmetrical (generally very unlikely) tree (Supplementary material 1). The matrix also had a strong phylogenetic signal, as conveyed by the g1 statistic and the PTP test (Supplementary materials 2 and 3).

Pairwise p-distances of MT-CO1 gene sequences between adult and larvae samples ranged from 0.1% to 1.4%, whereas among the larvae, the distance was 1.3%. Similarly, p-distances among *O. baylisi* sequences ranged from 0.4% to 1.1%, whereas the interspecific p-distances among *Ophidascaris* species sequences ranged from 5.6% to 10.4% (Supplementary material 4). The best-fit model, calculated via SMS in PhyML, under AIC, was GTR + R (Free Rate model), with four free rate categories, resulting in an ML tree with lnL = −4686.911054 score. The best-fit models, calculated via AMS in PAUP*, under BIC, were TrN + I for the first codon position, F81+I for the second codon position, and HKY + G for the third codon position. After 25% burn-in removal, MCMC samplings resulted in lnL = −4368.7859 mean estimated marginal likelihood (standard error = 0.0709; median = −4368.468). The ESS values were robust for all parameters.

**Fig. 6:**
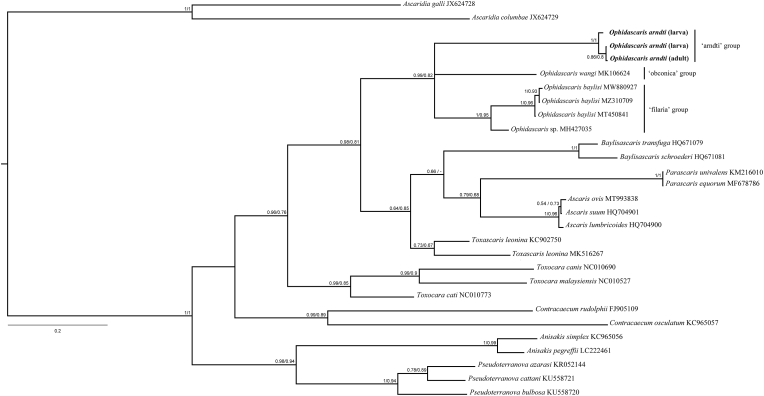
Phylogenetic relationships Bayesian tree based on partial MT-CO1 gene sequences of ascaridoid specimens, including *Ophidascaris* and outgroup. The numbers at the nodes are BPPs (left) and aLRT (right). The scale bar represents the number of substitutions per site.

The tree topologies recovered in our phylogenetic analyses were largely congruent (Fig. 6, Supplementary materials 5–7). The MT-CO1 sequences from adult samples and two larvae formed a highly supported monophyletic group in both ML and BI trees (aLRT = 1.00, ML-BP = 1.00, BPP = 1.00). This group formed a moderately to well-supported clade with sequences of other *Ophidascaris* species, depending on the tree (aLRT = 0.82, ML-BP = 0.64, BPP = 0.99), thus forming the genus *Ophidascaris*. This genus was branched into three main lineages, the ‘arndti’, ‘filaria’, and ‘obconica’ groups. The ‘filaria’ group was a well-supported clade (aLRT = 0.95, ML-BP = 0.90, BPP = 1.00), constituted by sequences of the monophyletic *O. baylisi* (aLRT = 0.96, ML-BP = 0.93, BPP = 1.00) and an unidentified species of *Ophidascaris*. The ‘obconica’ group was represented by just one sequence of *O. wangi*. The ‘arndti’ and ‘filaria’ groups formed a clade only in ML, although moderately supported (aLRT = 0.55, ML-BP = 0.60). In the BI, the three groups of *Ophidascaris* were in a polytomy. Within the superfamily Ascaridoidea, the genus *Ophidascaris* was sister to a clade formed by representatives of the family Ascaridade, forming a monophyletic group poorly supported by ML-BP = 0.35 and well-supported by aLRT = 0.81, and BPP = 0.98.

## 4 Discussion

According to Anderson (2000), the genus *Ophidascaris* parasitizes snakes, lizards, and occasionally amphibians. The genus is characterized by genital tubes restricted to the posterior region of the body in both sexes, females with two uterine branches, and the presence of interlabia. Freitas (1968) published an extensive review of the genus *Ophidascaris,* pointing to questions and clarifying systematic issues related to all species described. Sprent (1988) also revised and divided the genus into species groups, synonymized some species, and placed some of them as *species inquirenda*. Nevertheless, his division was not widely accepted. Vicente et al. (1993), Panizzutti et al. (2003), and Siqueira et al. (2005) still considered *O. travassosi*, *O. sprenti*, and *O. sicki* as valid species.

Comparing the present adult worms with other specimens of *O. arndti* placed in the ‘arndti’ group and synonymized with *O. arndti* by Sprent (1988), the morphological diagnostic characteristics were compatible with the species description. However, there is great morphometric variation among the several synonymized species, including our specimens, although congruent with morphometric data of the specimens originally described by Sprehn (1929) (Table 1). In particular, the number of precloacal papillae observed in our specimens and the specimens described by Sprehn (1929) was nearly the same (22 *versus* 20), as was the length of spicules (1.4–1.9 *versus* 1.6–1.7) and the pattern of distribution of postcloacal papillae. Sprehn (1929) described five pairs of postcloacal papillae distributed as follows: one pair laterally to the cloaca (paracloacal), two pairs located in the middle ventral region, and two pairs located laterally. We observed the same pattern in our specimens. Additionally, we detected the phasmids situated distally.

**Table 1:** Morphometric and other characteristics of *Ophidascaris arndti* (*sensu*Sprent, 1988).

Species	*O. arndti*Sprehn (1929)		*O. arndti* (syn. *O. travassosi*Vaz, 1938)		*O. arndti* (syn. *O. sprenti*Araujo, 1969)		*O arndti* Present study	
	Male	Female	Male	Female	Male	Female	Male	Female
Length (L)	24.2–37.2	27–48.2	50–60	70–80	59–66	81–101	9.13–17.77	30.75–58.98
Width	0.5–0.6	0.6–0.8	0.7	0.6	0.7–0.9	1.2–1.4	0.29–0.34	0.567–0.569
Labium	0.35 × 0.35		–	–	–	–	0.07–0.30 × 0.08–0.28	0.14–0.19 × 0.22–0.29
Interlabia	–		–		–		present	
Esophagus (E)	3.0		3.0	3.6	2.5–4	3.5–5	1.07–2.06	1.95–3.46
Proportion E/L (%)	8.1–12.4		5–6	4.5–5.1	3.1–6.1	4.3–5.0	11.6–11.7	5.7–6.3
Nerve ring			–	–	–	–	0.156	0.42–0.69
Excretory pore			–	–	–	–	0.88–1.03	0.56–1.93
Spicule (Spl)	1.6–1.7		2	–	2.82–2.88	–	1.44–1.92	–
Proportion Spl/L (%)	4.7–6.6	–	3.3–4.0	–	4.4–4.8	–	10.8–15.8	–
Precloacal papillae (pair)	20		±30	–	39	–	22	–
Paracloacal papillae (pair)	1		–	–	–	–	1	–
Postcloacal papillae (pair)	4		7	–	5	–	4	–
Tail	0.8–1.0	0.3–0.5	0.24	0.22–0.24	0.14–0.19	0.48–0.76	0.18-0-30	0.40–046
Vulva DAE	–	Middle third 28	–	Middle 33	–	Middle third 25–39	–	Anterior third 10.63–11.37
Egg length	–	0.070–0.087 × 0.061–0.070	–	0.064–0.068 × 0.058–0.062	–	0.068–0.093 × 0.058–0.074	–	0.076–0.084 × 0.060–0.070
Host	***Bothrops* [as *Lachesis*] *lanceolatus* possibly** ***B. jararaca* or *B. jararacussu***		***Crotalus durissus* [as *Crotalus terrificus*]**		***Crotalus durissus* [as *Crotalus durissus terrificus*]**		***Bothrops fonsecai***	
Locality	**Brazilian snake in the Berlin Aquarium**		**São Paulo, Brazil**		**Brazil**		**Rio de Janeiro, Brazil**	

DAE distance from the anterior end.

Li et al. (2014) described *O. wangi* found in the king rat snake *Elaphe carinata* (Günther, 1864) and added new morphological characteristics to *O. najae* (Gedoelst, 1916) found in the king cobra *Ophiophagus hannah* (Cantor, 1836) (Serpentes: Elapidae) in China. Li et al. (2014) emphasized that *O. tuberculatum* Siqueira et al., (2005) should be a junior synonym of *O. arndti* as they share similar morphologies, despite the lack of morphological studies comparing these two species. However, *O. tuberculatum* seems to be a distinct species even with deficient morphological characterization. *Ophidascaris tuberculatum* has longer spicules than *O. arndti* (2.45–2.90 × 1.81–1.91), one unpaired precloacal papilla and one postcloacal, female with conspicuous post-anal, muscular tubercle-like protuberance with a rugose surface, and longer body length (Siqueira et al., 2005). Undoubtedly, *O. tuberculatum* should be considered a *species inquirenda* until a rigorous redescription is carried out and, if valid, should be placed in the ‘arndti’ group. Moreover, Li et al. (2014) suggested that *O. durissus* Panizzutti et al., (2003) should belong to the genus *Hexametra* Travassos, 1920, due to the absence of interlabia.

*Ophidascaris sprenti*Araujo (1969) was synonymized with *A. arndti* by Sprent (1988). Although described with five pairs of postcloacal papillae, this pattern of postcloacal papillae may be considered consistent with *O. arndti*, as Araujo (1969) considered one paracloacal pair and four postcloacal pairs, as a whole. Moreover, *O. travassosi* Vaz (1938), also considered a junior synonym of *A. arndti* by Sprent (1988), has a different pattern and number of postcloacal papillae. The number and arrangement of paracloacal and postcloacal papillae are considerably stable in *Ophidascaris* spp. (Li et al., 2014). Therefore, the taxonomic contradictions may result from the different denominations for papillary configuration within the ‘arndti’ group.

Our adult specimens were similar to those described by Sprehn (1929). The length of the spicules was morphometrically close, although the proportions were different. Li et al. (2014) considered that the length of the esophagus and the spicules vary depending on the specimen age and body size, and thus should be used together with the ratio to the body length. Indeed, these characteristics are useful to identify ascaridoid nematodes, and this difference could be due to the smaller size of our specimens, probably in an early stage of development. Thiengo et al. (2010) showed that the length of spicules does not differ among different stages for *Angiostrongylus cantonensis* (L5 and adult), suggesting that it is one of the first characteristics to develop.

The life cycle of some species of *Ophidascaris* has been reported in studies of experimental infection by different authors (Ortlepp, 1922; Sprent, 1970; Araujo and Machado, 1980). We presented the first report of natural infection by this helminth's larvae in a wild intermediate host. In this study, the presence of third-stage larvae in the muscles of the montane grass mouse *A. montensis* indicates that this rodent may be an intermediate host of *O. arndti* in the Atlantic Forest of the state of Rio de Janeiro, given its high abundance, relative to the other rodents in the trapping areas (Cardoso et al., 2018). *Akodon montensis* is widespread along the South and Southeast Atlantic coast of Brazil, from the state of Rio de Janeiro and east of the state of Minas Gerais to the state of Rio Grande do Sul, and in eastern Paraguay and northeastern Argentina (Pardiñas et al., 2015). This species is quite abundant and one of the most frequently trapped in small mammal surveys within its geographic range (Pardiñas et al., 2015). This rodent is strictly terrestrial, which may favor the acquisition of eggs within the soil. Moreover, *B. fonsecai* is also terrestrial and its diet is specialized in small mammals (Martins et al., 2002). This species is restricted to mountain ranges up to a maximum of 1700 m a.s.l., in the states of Rio de Janeiro, São Paulo, and Minas Gerais (Nogueira et al., 2019), overlapping its distribution with *A. montensis* which, in the state of Rio de Janeiro, is found only above 800 m a.s.l. (Geise et al., 2001)*.* However, there is a lack of information concerning *B. fonsecai* population status. Araujo and Machado (1980) concluded in their study on *O. trichuriformis* life cycle that snake infection occurs by ingestion of anuran amphibians or other animals infected with third-stage larvae. Sprent (1988) postulated that *O. arndti* is transmitted to crotaline snakes when they feed on infected rodents. This was based on preliminary observations from an experimental study by Araújo and Sprent (unpublished), which indicated that, when eggs of *O. arndti* were fed to mice, larvae became encapsulated in the liver, and/or free and encapsulated in the scrotal sacs.

Our molecular analyses were crucial to identify the larvae at the species level because the immature nematode lacks specific diagnostic morphological characteristics. The MT-CO1 gene sequences of adult and larval nematodes formed a highly supported monophyletic group in all phylogenies and had low pairwise genetic distances, suggesting that these larvae and adult nematodes are conspecific. This group formed a polytomy with the sequence representing the ‘obconica’ group, and the clade formed by sequences representing the ‘filaria’ group. Thus, our results supported the ‘arndti’, ‘filaria’, and ‘obconica’ groups as independent lineages. More sequences of these groups and other *Ophidascaris* species groups would be necessary to further investigate the consistency of Sprent's species groups. Our results also supported the allocation of *Ophidascaris* to the family Ascarididae, although belonging to an early offshoot.

## Declaration of competing interest

The authors declare that they have no known competing financial interests or personal relationships that could have appeared to influence the work reported in this paper.
